# Beneficial perspective on *Staphylococcus epidermidis*: a crucial species for skin homeostasis and pathogen defense

**DOI:** 10.3389/fimmu.2025.1674392

**Published:** 2025-10-22

**Authors:** Ping Qi, Feiyu Gong, Min Leng, Zairong Wei

**Affiliations:** ^1^ Department of Burns and Plastic Surgery, Affiliated Hospital of Zunyi Medical University, Zunyi, China; ^2^ The 2011 Collaborative Innovation Center of Tissue Damage Repair and Regeneration Medicine, Affiliated Hospital of Zunyi Medical University, Zunyi, China; ^3^ The Collaborative Innovation Center of Tissue Damage Repair and Regeneration Medicine, Zunyi Medical University, Zunyi, China

**Keywords:** *Staphylococcus epidermidis*, coagulase-negative staphylococci, pathogenicity, commensal, skin homeostasis

## Abstract

Human skin harbors a diverse microbiome that shapes immune function, protects against pathogens, and sustains tissue homeostasis. Among its dominant members, *Staphylococcus epidermidis*—a coagulase-negative staphylococcus—was long considered primarily an opportunistic pathogen, especially in the context of biofilm formation and implant-associated infections. However, emerging evidence reframes *S. epidermidis* as an active commensal, capable of controlling inflammation, supporting antimicrobial defenses, and stabilizing the cutaneous barrier. These dual roles are largely determined by its extensive strain-level heterogeneity and dynamic colonization strategies. Here, we review current progress in understanding the ecological versatility of *S. epidermidis*, with particular focus on its potential benefits, its diversity and colonization dynamics, and the balance of costs and benefits associated with its presence on human skin.

## 1 Introduction

The human skin is a complex physiological barrier that plays a critical role in maintaining internal homeostasis and defending against pathogens. The skin is colonized by commensal bacterium and serves as a physical barrier preventing pathogen invasion. It is home to a diverse community of resident commensal flora, including bacteria, archaea, fungi, and viruses (1). Similar to gut microorganisms, skin microorganisms have essential roles in protecting against invading pathogens, educating our immune system, and breaking down natural products (2). The skin, including its appendages, boasts a surface area of at least 30 m^2^, surpassing even the gut’s surface area (3). In April 2023, the U.S. Food and Drug Administration recently approved Vowst, a fecal microbiota product taken orally, for the prevention of recurrent *Clostridioides difficile* infection. This groundbreaking approval highlights the potential benefits of microbiota products in treating and preventing infections by restoring the natural balance of microorganisms in the gut. The skin microbiome comprises a diverse array of commensal organisms with distinct, and sometimes opposing, effects on host physiology. For example, *Staphylococcus hominis* produces lantibiotics that inhibit *Staphylococcus aureus* (*S. aureus*) colonization, demonstrating the protective capacity of closely related staphylococci (4). Conversely, while *Cutibacterium acnes* contributes to lipid metabolism in sebaceous regions, it is also strongly implicated in acne pathogenesis when dysregulated (5, 6). Members of *Corynebacterium* spp. can educate skin-resident T cells and support immune homeostasis, yet certain strains act as opportunistic pathogens, particularly in immunocompromised hosts (7, 8).

Against this backdrop, *S. epidermidis* stands out as a particularly versatile commensal. Unlike *C. acnes*, it rarely contributes to chronic inflammatory diseases, and unlike *Corynebacterium*, its commensal bacterium activities are more consistently observed across different strains. Beyond the antimicrobial function of *S. hominis*, *S. epidermidis* additionally promotes barrier integrity, modulates both innate and adaptive immunity, and secretes biofilm-inhibitory molecules that limit pathogen expansion. These attributes highlight its unique potential as a keystone commensal and position it as a rational candidate for probiotic-based interventions aimed at improving skin health. As a dominant commensal of the skin, *Staphylococcus epidermidis* exhibits remarkable strain-level diversity that underpins its dual capacity to promote skin health or contribute to pathology. This Review examines the ecological and functional diversity of *S. epidermidis*, critically assesses the benefits and risks of its colonization, and highlights future directions for advancing research on the skin microbiome.

## 2 Current experimental approaches in microbiome

Current methodologies for studying bacteria each possess distinct strengths and limitations. Traditional culture-based approaches, while foundational, often underestimate the true diversity of microbial communities due to their reliance on artificial growth conditions (9). To address this limitation, molecular techniques such as 16S rRNA gene sequencing for bacteria and ITS1 sequencing for fungi have become widely adopted (10, 11). These approaches target conserved genetic markers to achieve more precise microbial identification, reducing the biases inherent in culture-dependent methods and providing more comprehensive tools for investigating the skin microbiome.

Among these, 16S rRNA sequencing remains a widely used strategy for profiling microbial communities, valued for its cost-effectiveness and robust performance at the genus level. However, its taxonomic resolution is limited and susceptible to factors such as primer selection and variations in rRNA gene copy numbers (12). In contrast, shotgun metagenomics offers higher resolution and functional insights by directly sequencing all genetic material, but the sequencing depth required for meaningful analysis often entails substantial costs. Shallow shotgun sequencing has emerged as a practical compromise, providing improved taxonomic resolution and reduced technical variation compared to 16S methods (13). Recent advances, including spatial transcriptomics and single-cell sequencing, have further expanded the methodological repertoire for microbiome research. Single-cell sequencing enables the characterization of gene expression heterogeneity and the functional states of individual microorganisms (14), while spatial transcriptomics provides crucial anatomical context by mapping microbial distributions within tissues (15). Together, these technologies offer complementary perspectives, enriching our understanding of microbial ecology.

## 3 Dual roles of *S. epidermidis*

The *Coagulase-negative staphylococci* (*CoNS*) are among the most abundant colonizers of all skin sites. Two decades ago, Kloos and Bannerman (16) updated our understanding of the clinical significance of *CoNS*, defining them by their distinction from *coagulase-positive staphylococci*. *Coagulase-positive organisms*, such as *S. aureus*, are known to cause a wide range of infections and are considered universal pathogens (17, 18). However, a more recent study revealed that *Staphylococcus lugdunensis (* 19) can inhibit the growth of *S. aureus* through production of a new antibiotic called legumin, a cyclic peptide containing thiazolidine. While this phenomenon may seem counterintuitive, it underscores the complex interplay between the host and microbiota.

*S. epidermidis*, as a major representative of *CoNS*, has garnered considerable attention in recent years due to its evolving role, shifting from a “conditional pathogen” to a “commensal”. While historically recognized as a primary causative agent of healthcare-associated infections, it is now also appreciated as a crucial commensal bacterium in maintaining skin homeostasis, exhibiting multifaceted roles in skin immune regulation, infection defense, and wound repair. Notably, as the predominant *CoNS* species within the skin microbiome, *S. epidermidis*, as a major species in the skin microbiome, has often been considered an opportunistic pathogen in the past, causing a series of nosocomial infections (20), particularly in immunocompromised individuals or those with implanted medical devices (21). Of particular concern is the fact that S. epidermidis is a leading cause of infections associated with indwelling medical devices, including peripheral and central intravenous catheters, resulting in significant medical and economic burdens (22). Furthermore, up to 20% of patients with cardiac devices can develop infections, leading to erythema, pain, purulence around the site of the infection, and potentially life-threatening sepsis (23). *S. epidermidis* is a common cause of bacteremia in preterm infants, as established by researchers (24). Clinically relevant neonatal mouse models have been developed to study the combined effects of bacterial infection and subsequent hypoxic-ischemic brain injury (25). Immunization of mice with a PIA-rSesC conjugate vaccine has been shown to protect against *S. epidermidis* infection (26). *S. epidermidis* employs several mechanisms to survive and cause infections. These include the ability to form biofilms (27, 28) on medical devices and host tissues, which provides a physical barrier against the host immune system.

In systemic disease states such as diabetes, immunosuppression, and chronic inflammation, *Staphylococcus epidermidis* colonization patterns and pathogenic mechanisms undergo significant alterations. Compromised skin barrier function, as seen in atopic dermatitis, can lead to *S. epidermidis* overgrowth, exacerbating inflammatory responses by upregulating barrier-disrupting genes and increasing epidermal dye penetration (29). Under these conditions, commensal-specific CD4+ T cells are predisposed to differentiate into effector T cells rather than regulatory T cells, fostering a chronic wound-like inflammatory milieu (30). In lymphedema models, *S. epidermidis* colonization amplifies pre-existing skin barrier dysfunction (31). During immunosuppression, *S. epidermidis* can transition into an opportunistic pathogen by acquiring virulence factors from *S. aureus* or evolving into methicillin-resistant strains (32). Microbial metabolites such as short-chain fatty acids participate in immune regulation by modulating keratinocyte activity (33). However, in pathological states, transcriptomic reprogramming may drive a shift from a commensal to a pathogenic phenotype (34).

**Figure 1:**
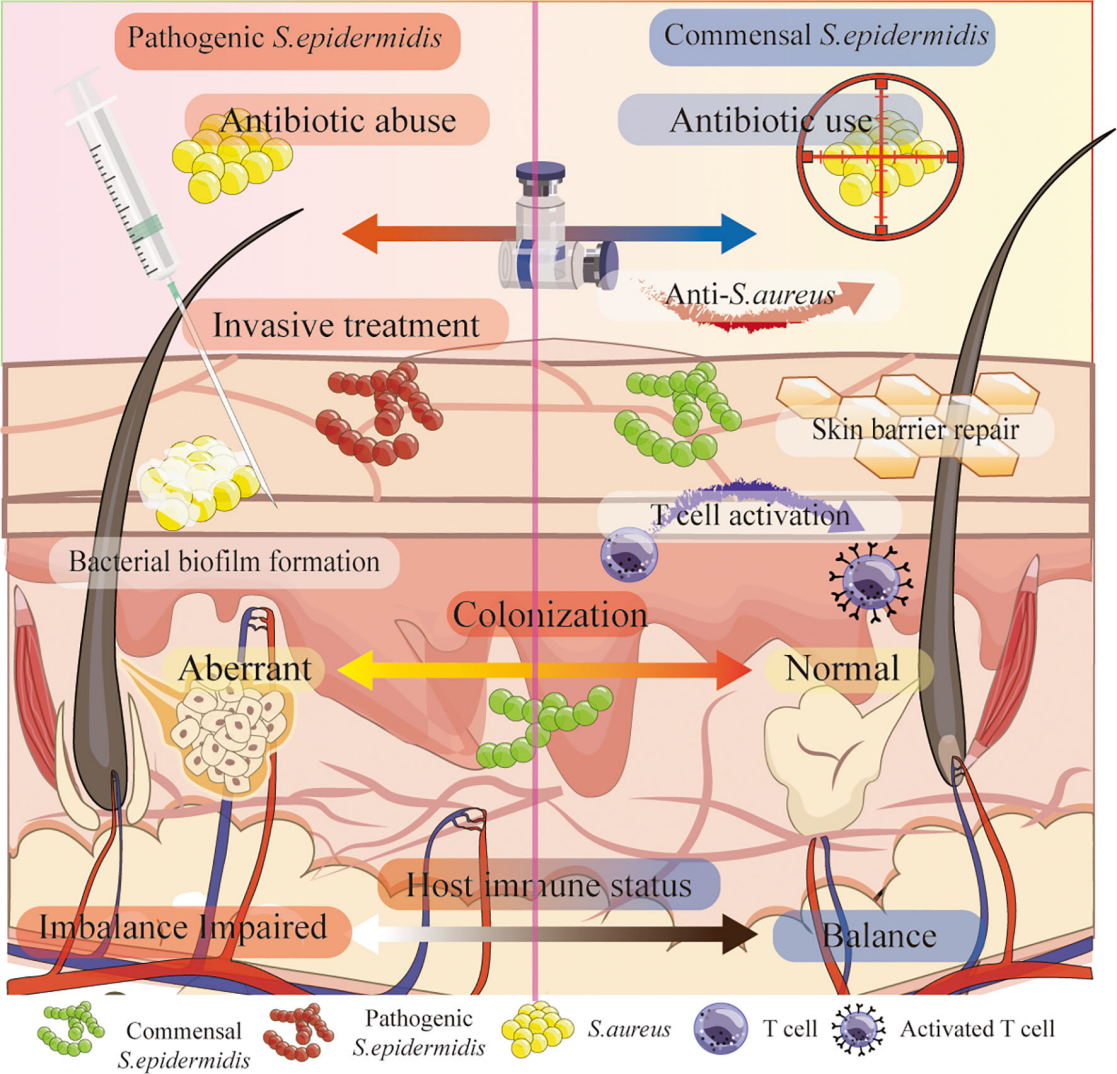
Dual roles of *S. epidermidis* on the skin. Under conditions such as antibiotic misuse, invasive procedures, or compromised immune function, *S. epidermidis* can exhibit pathogenic traits (left panel) (36, 37) including biofilm formation and dysregulated colonization (29). Conversely, commensal *S. epidermidis* (right panel) contributes to host defense by inhibiting *S. aureus*, promoting skin barrier repair (38), and activating T cells (39). The host’s immune status and colonization dynamics ultimately determine whether *S. epidermidis* behaves as a commensal or an opportunistic pathogen.

In recent years, research has increasingly focused on exploring the beneficial attributes of *S. epidermidis* as a commensal skin microorganism. The organism’s advantageous role as a skin commensal has received considerable attention within the scientific community. Several studies (35) Suggest that *S. epidermidis* may offer benefits in wound healing and infection defense. Accumulating evidence underscores the clinical relevance of *S. epidermidis*, both as a potential commensal probiotic and as a therapeutic target in dermatology in **Figure 1**. Neonatal colonization with *S. epidermidis* exerts long-lasting immunological effects by inducing regulatory T cells and promoting tolerance to commensal antigens. Clinical observations link aberrant colonization patterns – such as those seen in infants delivered via cesarean section – to an increased risk of atopic dermatitis, suggesting that early-life microbial exposure can shape lifelong skin health (40).

In therapeutic contexts, *S. epidermidis*-based interventions have demonstrated promise in inflammatory skin diseases, including atopic dermatitis. Clinical studies report that combining live biotherapeutics derived from *S. epidermidis* with topical corticosteroids enhances treatment outcomes by suppressing pathogenic *S. aureus* overgrowth and modulating cutaneous immune responses (41). Collectively, these findings support the view that *S. epidermidis* is not only a key ecological regulator of the skin microbiome but also a viable candidate for translational strategies aimed at restoring microbial balance and improving clinical outcomes. Therefore, this review focuses on an in-depth exploration of the beneficial roles of *S.epidermidis* in immune regulation, antimicrobial defense, and barrier repair, highlighting its potential to enhance skin health and effectively prevent infections.

## 4 Immune regulation mechanisms

### 4.1 Immune balance and host interaction

*S. epidermidis* typically engages in a mutually commensal relationship with the host, contributing to infection prevention and the production of antimicrobial peptides (42). As a commensal bacterium, *S. epidermidis* colonization not only modulates the innate immune response but also contributes to the development and priming of the adaptive immune system. Specifically, it induces neutrophil CXCL10 signaling in skin wounds, which recruits type I interferon-producing plasmacytoid dendritic cells and drives T cell-independent wound repair (43). Further supporting the protective role of *S. epidermidis*, Murphy et al. (44) reported that a specific strain of *S. epidermidis*, isolated from healthy human prostatic secretions, can modulate immune responses to reduce inflammation and pain in a murine model of chronic prostatitis. *S. epidermidis* primes CD8+ T cells, which serve a dual purpose in cutaneous immunity and wound healing (45). Studies of gnotobiotic mice have demonstrated that *S. epidermidis* skin colonization is essential for effector T cell development and function, as well as for the early localization and priming of mucosal-associated invariant T cells (39), which are an important component of nonclassical cutaneous immune signaling.

### 4.2 Adaptive immunity enhancement

*S. epidermidis* enhances adult skin barrier immunity through a coupled mechanism between dendritic cells and T cells. It activates non-inflammatory skin-resident CD11B+ dendritic cells, which induce IL-17A+ CD8+ T cell homing to the epidermis and enhance barrier immunity against opportunistic pathogens like *S. aureus (* 46). IL-1β serves as a central mediator in host defense and immune homeostasis. *Staphylococcus epidermidis* induces keratinocytes to express and release mature IL-1β, thereby activating innate immune responses that help maintain skin barrier integrity and suppress opportunistic pathogens (38). This process is primarily mediated through TLR2 signaling, which not only triggers IL-1β production but also promotes the expression of antimicrobial peptides such as β-defensin-3, and often synergizes with TGF-α to enhance skin protective functions (47). To prevent excessive inflammation, *S. epidermidis* concurrently induces host regulatory proteins, such as A20, which inhibit NF-κB signaling. This limits the overproduction of IL-1β and antimicrobial peptides, ultimately maintaining microbial homeostasis (48). Notably, the host IL-1β response to *S. epidermidis* is more subdued compared to the response to pathogenic *S. aureus*. This allows the host to differentiate between commensal and pathogenic bacteria, fostering immune tolerance and commensal coexistence (49).

*S. epidermidis*-induced CD8+ T cells also promote re-epithelialization of the skin after injury, accelerating wound closure. Furthermore, *S. epidermidis* activates γδT cells and upregulates perforin-2 (50) an antimicrobial protein that kills intracellular bacteria – in human skin ex vivo in a cell-specific manner. Perforin-2 upregulation following *S. epidermidis* stimulation correlates with an increased ability of skin cells to kill intracellular *S. aureus* (51, 52). Besides, Strbo et al (53). demonstrated that *S. epidermidis* facilitates the clearance of intracellular pathogens by upregulating antimicrobial proteins such as perforin-2 in skin γδT cells. In neonatal skin development, there is a critical time window required for establishing tolerance to commensal microorganisms while maintaining a discrete response to pathogens (49, 54). Additional research suggests that *S. epidermidis* may play a key role in reducing IL-33 and Th2 inflammation by blocking allergen-induced cellular necroptosis in allergic nasal epithelium (55), which promotes skin-homing T cells to produce cytokines that contribute to host defense and skin inflammation.

### 4.3 Inflammation regulation

Lipoteichoic acid from distinct *S. epidermidis* strains lessened the generation of TNF and IL-6 in response to skin harm through Toll-like receptor 3(TLR3), inhibiting both inflammatory cytokine release from keratinocytes and inflammation triggered by injury through a TLR2-dependent mechanism (56). “Direct evidence for TLR3’s involvement in the commensal relationship with *S. epidermidis* remains limited, but it likely plays a modulatory role in IL-1β-mediated inflammation. TLR3 expression, influenced by interferon or microbial signals, may fine-tune IL-1β release, thereby preventing excessive inflammation (57). While *S. epidermidis* primarily signals through TLR2 to activate NF-κB and induce the production of IL-1β and antimicrobial peptides (58), potential TLR3 interactions could further balance this response. This coordinated signaling ensures effective host defense, maintains skin barrier integrity, and supports commensal coexistence. Li (59) identified a formerly unidentified lipopeptide 78 (LP78) in *S. epidermidis* and showed that LP78 repressed TLR3-mediated skin inflammation, leading to improved wound healing.

In sum, LP78 derived from *S. epidermidis* obstructs skin inflammation and is indicative of being a prospective element in treating refractory or non-healing injuries. The immune response modulation by *S. epidermidis* is also highlighted in transcriptomic studies. Masters (60) demonstrated that infections caused by *S. epidermidis* elicit specific gene expression profiles, notably involving cytokines such as IL13, IL17D, and MMP3, which are elevated during *staphylococcal* infections. This suggests that *S. epidermidis* may influence host immune responses, potentially contributing to its beneficial role in maintaining skin homeostasis and preventing pathogenic colonization. Key commensal mechanisms of *S. epidermidis* are summarized in **Table 1**.

**Table 1:** Protective mechanisms employed by *S. epidermidis* to maintain skin homeostasis.

Factor	Description	Function	Reference
CXCL10	Recruit type I interferon-producing plasmacytoid dendritic cells and drive T cell-independent wound repair	Drive wound repair	(43)
CD11B+ dendritic cells	Induce IL-17A+ CD8+ T cell homing to the epidermis and enhance barrier immunity	Against opportunistic pathogens	(46)
TLR2	promotes the expression of β-defensin-3	Main skin functions	(48)
NF-κB	limits the overproduction of IL-1β and antimicrobial peptides	Maintaining microbial homeostasis	(48, 49)
Perforin-2	Upregulates perforin-2 expression in human skin	An increased ability to kill *S. aureus* Clearance of intracellular pathogens	(50–53)
Lipoteichoic acid	Selectively on keratinocytes triggered through TLR3	Inhibits inflammatory	(56)
Lipopeptide 78	Represses TLR3-mediated skin inflammation	Improves wound healing	(59)
GluSE	Degrades proteins that are crucial for *S. aureus* biofilm formation and host epithelial adhesion.	Inhibits *S. aureus* biofilm formation	(61–64)
PSMγ, PSMδ	Cooperatively inhibits the growth of S. aureus and *Group A Streptococcus*	Inhibits pathogen growth	(65–67)
6-TG	Inhibit purine biosynthesis and toxin production	Suppresses S. aureus	(68)
Epilancins A37	Induces the formation of intracellular membrane vesicles, which are heavily loaded with the compound and are essential for the antibacterial activity of the epilancin	Selectively inhibit natural competitors	(69)
Epifadin	A non-ribosomally synthesized antimicrobial peptide composed of both proteinogenic and non-proteinogenic elements	Disrupting bacterial membranes	(70)

## 5 Antimicrobial compounds

The first organisms reported to inhibit *S. aureu*s originated from a subset of *S. epidermidis* strains that express the serine protease glutamyl endopeptidase (61) (Esp; also known as GluSE). The Esp produced by these *S. epidermidis* strains degrades proteins that are crucial for S. aureus biofilm formation and host epithelial adhesion. *In vivo* studies have shown that Esp-secreting *S. epidermidis* eliminates *S. aureus* nasal colonization (62). Additional research suggests that the resulting bactericidal activity of β-defensin induced by *S. epidermidis* was sufficient to kill *S. aureus* within biofilms (48). Epidemiological studies have demonstrated that the presence of Esp-secreting *S. epidermidis* in the nasal cavities of human volunteers correlates with the absence of *S. aureus* (63). These findings strongly suggest that microorganisms originating from a subset of S. epidermidis strains expressing Esp effectively inhibit *S. aureus* biofilm formation, holding significant potential for the treatment and prevention of biofilm-related infections (64).

Small compounds produced by *S. epidermidis* directly influence innate immune receptors. *S. epidermidis* produces phenol-soluble modulins (PSMs), a family of small, amphipathic α-helical peptides abundant on the normal epidermis and in hair follicles (65). Recent studies have demonstrated that PSMs can synergize with host antimicrobial peptides to enhance the killing of the pathogen Streptococcus *pyogenes*. *S. epidermidis* and *Staphylococcus hominis* have been shown to produce novel antibiotics that can synergize with the human cathelicidin antimicrobial peptide LL-37 and inhibit the growth of S. aureus (66). Specifically, both PSMγ and PSMδ exhibit cooperative activity with the host antimicrobial peptide LL-37, resulting in enhanced antimicrobial action against S. aureus and *Group A Streptococcus*. The synthesis of PSMγ involves an assembly line composed of a multimodular PKS, whose gene cluster encodes an enzyme system responsible for forming the γ-dihydropyrone backbone structure. The synthesis of PSMδ may follow a similar mechanism (71, 72).Furthermore, *S. epidermidis* PSMγ has been detected in the epidermis and dermis of normal human skin and has been shown to reduce GAS survival in pretreated mouse skin wounds (67).

**Figure 2:**
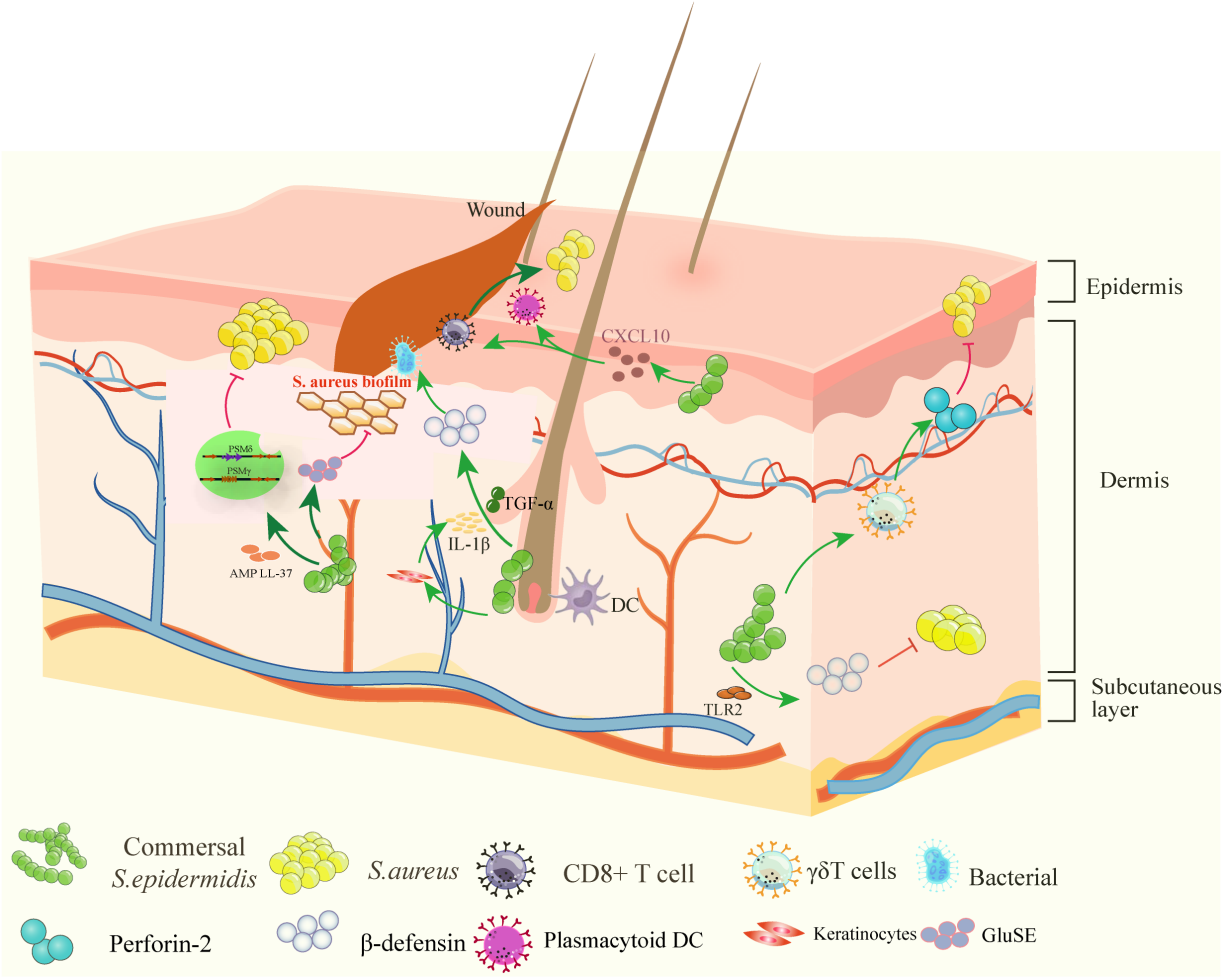
The ubiquitous skin commensal *S. epidermidis* positively impacts to barrier homeostasis and integrity. *S. epidermidis* promotes skin health through various mechanisms: TLR2 activation induces IL-1β and TGF-α production, stimulating antimicrobial peptide expression β-defensin (38, 47, 48); CXCL10 signaling recruits type I interferon-producing plasmacytoid dendritic cells (43), driving T-cell-independent wound repair; PSMγ/δ synergizes with host LL-37 to enhance killing of *S. aureus* and *streptococci* (65); CD8+ T cell proliferation accelerates re-epithelialization and clearance of intracellular pathogens (45); perforin-2 upregulation in γδ T cells strengthens antibacterial defenses (50). These factors collectively establish a multi-layered protective network; PSMγ and PSMδ synergize with the host peptide LL-37 to enhance antimicrobial activity against *S. aureus (* 67); GluSE degrades essential factors for *S. aureus* biofilm formation and epithelial adhesion (62). TLR2, Toll-like receptor 2; IL-1β, interleukin-1β; TGF-α, transforming growth factor α; PSM, phenol-soluble modulins; GluSE, glutamyl endopeptidase.

*S. epidermidis* produces the purine analog 6-thioguanine (6-TG), which suppresses *S. aureus* growth by inhibiting purine biosynthesis and toxin production (68). *S. epidermidis* both produces lantibiotics (e.g., epilancin A37, Pep5) and is a common susceptible bacterium. The lantibiotics it produces play a key role in microbial competition. Bioinformatic analyses have also revealed Epilancins, a family of antimicrobial peptides widely encoded in *staphylococcal* genomes. In *S. epidermidis* A37, Epilancins have been shown to selectively inhibit natural competitors such as *Corynebacterium* spp (69). The S. epidermidis strain KSE112 produces the antibiotic Pep5, which exhibits potent activity against *S. aureus* (73), as shown in **Figure 2**.

## 6 Translational and therapeutic perspectives

Having established the molecular and cellular basis of *S. epidermidis’* role in skin homeostasis, preclinical and early clinical studies are exploring its potential, and that of its metabolites, as a live biotherapeutic, presenting both opportunities and challenges.

*S. epidermidis* releases specific antimicrobial peptides (bacteriocins) that selectively target *S. aureus*, a common isolate from the skin of patients with atopic dermatitis (AD). These S. aureus-inhibiting *CoNS* strains are rare in AD, but topical application of such strains has been shown to reduce S. aureus load *in vivo* (74). Reintroduction of antimicrobial *CoNS* strains to human subjects with AD also decreased S. aureus colonization (75). However, a primary challenge in developing *S. epidermidis* as a live biotherapeutic lies in distinguishing commensal from potentially pathogenic strains. Safety assessments must therefore focus on virulence markers, including biofilm formation capacity (76), presence of hemolysin genes (77), and presence of the staphylococcal cassette chromosome mec element (78). Suitable therapeutic candidates should lack DNase, gelatinase, and other virulence factors, a characteristic that can be verified via *in vitro* hemolysis assays (79). Comparative genomics have revealed significant inter-strain variation in immunomodulatory functions such as keratinocyte interaction and short-chain fatty acid production (33). Notably, the agr quorum sensing system type determines the capacity to inhibit S. aureus; Type I and IV strains effectively suppress S. aureus virulence factor expression and mitigate skin inflammation (80), while Types II and III lack this functionality and may exhibit hospital adaptation tendencies (81)]. Encouragingly, skin commensal strains exhibit distinct transcriptomic profiles compared to infection isolates (34), providing novel molecular markers for therapeutic strain selection.

Beyond strain selection, a key challenge for *S. epidermidis* live biotherapeutics is maintaining viability and stability during manufacturing and storage. Recent research has focused on controlled bacterial proliferation, rather than complete inhibition, by incorporating specific nutrient limitation factors into the formulation, allowing the strain to remain metabolically active but division arrested (82). This approach circumvents cold-chain shipping costs associated with traditional refrigeration and avoids the use of antibiotics or resistance markers (83). Given the correlation between *S. epidermidis’* therapeutic efficacy and its electroactivity, researchers are developing electroactivity-enhancing delivery systems. Hydrogels containing conductive polymers, such as polyaniline derivatives, have been shown to enhance electron transfer efficiency, improving the strain’s competitive inhibition of S. aureus (83). Guided by the ecological interactions within the skin microbiome, recent studies have also proposed “microbiota-guided” combination therapies. For example, combining *S. epidermidis* with specific ratios of Bacillus subtilis can form stable biofilm structures, significantly enhancing spatial exclusion of pathogens (84). This combination not only increases the persistence of individual strains but also activates a broader network of immune defenses through interspecies signaling (85). For biofilm-associated infections, composite formulations containing phage lysins and *S. epidermidis* are being developed, where the live bacteria exert immunomodulatory effects while the lysins specifically degrade the pathogen’s biofilm matrix (86, 87). This synergistic design addresses antibiotic resistance and reduces the immunogenic risk associated with using phage lysins alone. Crucially, such combination products require rigorous subspecies typing (e.g., 16S rRNA sequencing) and functional validation to exclude potentially pathogenic clones (88, 89).

Production of *S. epidermidis* live biotherapeutic products must adhere to strict GMP standards, with particular emphasis on strain quality control. The US FDA classifies skin microbiome products as biologics, requiring compliance with 21 CFR 610 and submission of strain genomic stability data (90, 91). The EU EMA incorporates live biotherapeutics within the Advanced Therapy Medicinal Product framework, emphasizing the maintenance of strain functionality during production (92). An adaptive regulatory pathway is recommended for *S. epidermidis* products: preclinical studies should focus on assessing strain safety (including screening for antibiotic resistance genes) (93), while clinical studies should use multi-omic markers of microbiome-host interactions as surrogate endpoints (94).

## 7 Conclusion and outlook

In immunocompromised patients (e.g., transplant recipients, chemotherapy patients, or individuals with HIV), *S. epidermidis* is more likely to breach host immune defenses and cause infection. The prevalence of methicillin-resistant *S. epidermidis* (*MRSE*) is a consequence of antibiotic overuse in hospital settings. *MRSE* is not only resistant to β-lactam antibiotics but may also carry multiple drug resistance genes, complicating treatment (95). Disruption of the normal skin microbiome, such as *S. aureus* overgrowth, can also diminish the commensal protection afforded by *S. epidermidis*. While *S. epidermidis* typically inhibits other pathogens by producing antimicrobial peptides like lantibiotics, pathogenic clones (e.g., HA-*MRSE*) can acquire virulence factors through horizontal gene transfer (96, 97). Pathogenic conversion of *S. epidermidis* results from a confluence of host immune status, environmental selective pressures (e.g., antibiotics, medical devices), and bacterial adaptation mechanisms (biofilm formation, antimicrobial resistance, and virulence genes) (34, 36).

The complex and multifaceted role of *S. epidermidis* in skin health underscores the critical importance of continued research into its host interactions, aimed at developing strategies to promote optimal skin health and prevent infection. This includes identifying symbiotic strains that may become pathogenic under specific conditions and elucidating the mechanisms by which *S. epidermidis* contributes to skin barrier development, maintains immune homeostasis, and suppresses opportunistic pathogens. Given the competitive dynamics within bacterial communities (98), growing evidence suggests that acne may arise from imbalances between *Cutibacterium acnes* (formerly *Propionibacterium acnes*) and *S. epidermidis (* 99). Further studies have demonstrated that glycerol fermentation by staphylococci represents an innate antimicrobial defense mechanism, potentially applicable in cosmetic formulations (100). Recent work also indicates that certain colonizers can trigger T cell responses, inducing preemptive adaptive immunity (101). Looking ahead, continued research into *S. epidermidis* is expected to contribute to novel therapeutic strategies, ranging from microbial products and probiotic interventions to the discovery of new antimicrobial agents derived from its metabolites. Collectively, these findings emphasize its dual nature – beneficial or detrimental depending on strain type, host factors, and environmental context – and call for deeper mechanistic insights to optimize its clinical utility in personalized medicine.

Despite these advances, several critical questions remain unresolved. First, the mechanisms underlying strain-specific variability are poorly understood. The remarkable heterogeneity of *S. epidermidis* accounts for its diverse pathogenic potential; however, the genetic drivers enabling certain lineages (e.g., ST2) to evolve into highly virulent pathogens, while others remain commensals, are incompletely characterized. Specifically, how virulence factors such as *icaAD* and *aap* are differentially expressed across strains remains unclear (102). Similarly, the regulation of biofilm-associated genes (e.g., the *ica* operon) under varying environmental conditions, such as oxygen tension, is incompletely defined (103, 104). The mechanisms linking biofilm formation to the persistence of multidrug resistance across strains also remain underexplored, limiting the development of targeted therapies (105). Parallel to addressing these biological questions, new methodological advances are essential. CRISPR interference screening has been applied for high-throughput identification of essential genes under diverse environmental conditions, revealing adaptive mechanisms and virulence gene functions (102). Scaling such approaches to metagenomic and spatial multi-omics analyses will enable higher-resolution tracking of strain-specific dynamics within the skin microbiome. Targeting biofilm-regulatory loci such as *ica* or *sdrG* with small-molecule inhibitors also represents a promising therapeutic avenue (106).

Looking ahead, several research directions deserve particular emphasis. First, a detailed mechanistic understanding of beneficial pathways is needed to define how immune activation, colonization resistance, and fermentation products like short-chain fatty acids contribute to skin barrier homeostasis (107). Second, the context-dependent roles of *S. epidermidis* in inflammatory skin diseases, such as atopic dermatitis, must be clarified, particularly how strain heterogeneity shifts commensal bacteria toward pathogenic behavior through proteases or phenol-soluble modulins (108). Third, systematic identification and validation of probiotic or biocontrol strains (e.g., SAS1), combined with optimized delivery systems such as topical formulations, could enable stable colonization and therapeutic efficacy (109). Fourth, distinguishing commensal versus pathogenic biofilms and developing selective anti-biofilm strategies will be critical for both infection control and the safe application of probiotics (110). Finally, tackling antimicrobial resistance—especially the rising prevalence of methicillin-resistant *S. epidermidis*—will require integrating microbiome research with antibiotic susceptibility testing to guide rational therapeutic design.

In summary, *S. epidermidis* presents a compelling paradox: a protective commensal and an opportunistic pathogen. Future research should integrate mechanistic insights, technological innovation (e.g., strain-level metagenomics, metabolomics, CRISPR-based functional genomics), and translational applications (engineered commensals, metabolite-based therapeutics) to unlock its full potential in dermatology. The combination of single cell sequencing and spatial transcriptomics, already valuable in gut microbiome studies for dissecting host interactions and disease mechanisms, holds great promise for application in the skin microenvironment. By addressing key questions surrounding strain heterogeneity, biofilm regulation, and resistance mechanisms, *S. epidermidis* can ultimately be harnessed as a novel probiotic and therapeutic tool while minimizing its inherent pathogenic risks, paving the way for precision microbiome-based therapies.
